# Chicago College of Pharmacy

**Published:** 1869-07

**Authors:** 


### CHICAGO COLLEGE OF PHARMACY.

---

### Special Meeting—New Members—Honorary Members—Election of Delegates.

A special meeting of the Chicago College of Pharmacy was held at their rooms, in Rice's Building, on the afternoon of the 24th June,—the President, E. H. Sargent, in the chair.

The proceedings of the previous meeting were read and approved.

The minutes of the meeting of the Board of Trustees were also read and approved.

The following new members were elected—James E. Marshall, Centralia, Ill.; George F. Layton, South Bend, Ind.; John C. Evans, New Albany, Ind.; F. M. Goodman, Ph. T. Hines, Edward Vincant, K. A. Hunter, Homer A. Smith, H. T. Patterson, Fred C. Webber, George C. Cunningham, Chicago.

The following foreign honorary members were, after discussion, elected—Dr. J. Attfield, Dr. T. Redwood, Henry

Deane, Daniel Hamburg, London, England; Henry B. Brady, Newcastle-on-Tyne, England; Dr. G. C. Wittsteing, Munich, Bavaria; Dr. H. Hager, Berlin, Prussia; Dr. Frederick Mohr, Bonn, Prussia; Dr. F. A. Fluckiger, Berne, Switzerland; Augustus Ambroise Delandre, Messrs. Robinet and Boullay, Paris; Dr. Arthur Casselmann, St. Petersburg, Russia; Dr. G. Dragendorff, Darpet, Russia.

Edward T. Schloetzer was elected a trustee of the college, to fill a vancancy occasioned by the absence from the city of Louis A. Diehl.

The following gentlemen were elected as delegates to represent the College at the Convention of American Pharmaceutical Association, to be held in this city on the 7th of September next—Albert E. Ehert, F. Mahla, James W. Mill, George M. Hambright, Thomas Whitfield.

The following delegates were appointed to represent the College at the Convention to Revise the National Pharmacopœia, to be held in Washington in May, 1870—Messrs. Albert E. Ebert, Henry Biroth, C. Louis Diehl. Alternates—James W. Mill, F. Mahla, Louis Strehl.

The meeting then adjourned.

